# Experiences of End-of-Life Care of Older Adults with Cancer From the Perspective of Stakeholdersin Iran: A Content Analysis Study

**DOI:** 10.31557/APJCP.2021.22.1.295

**Published:** 2021-01

**Authors:** Zohreh Ghezelsefli, Fazlollah Ahmadi, Eesa Mohammadi, Martine Puts RN

**Affiliations:** 1 *Department of Nursing, Faculty of Medical Sciences, Tarbiat Modares University, Tehran, Iran. *; 2 *Department of Nursing, Lawrence S. Bloomberg Faculty of Nursing, University of Toronto , Toronto, Ontario, Canada. *

**Keywords:** End of life, cancer care, geriatrics

## Abstract

**Objectives:**

To describe end-of-life care forolder adults with cancer admitted to the hospital in Tehran, Iranto determine if there were any gaps in care for older adultsthat can be improved.

**Materials:**

This study used a qualitative descriptive study design. In total, 37 individualsincluding patients, healthcare team members, and family caregivers, participated in the study. Semi-structured interviews using topic guides were conducted, and the thematic content analysis method described by Braun and Clarke (2006), was used to analyze the data.

**Results:**

In total, 37 Iranian participants (12 male and 25 female), including 14 nurses, 3 oncologists, 1 social worker, 1 chaplain, 1 psychologist, 11 family members and 6 patientsinterviewed.Our main themes of end-of-life carewere:1) barriers to providing and receiving quality care for families and patients; and 2) coping strategies and empowerment of families and patients.

**Conclusion:**

Healthcare providers are recommended to familiarize themselves with the burden faced by patients and family caregivers who take care of older adults with chronic diseases at home, and they should organize their supportive and consulting actions. In order to improve the quality of life of older patientsand their family caregivers.

## Introduction

Cancer is the second cause of death worldwide. The World Health Organization reported that 9.6 million people died of cancer in 2018 (World Health Organization Cancer, 2019), with the median age at death being 72 years (National Cancer Institute, 2019). This disease affects not only the patient, but also their family and society. Cancer patients, especially those in late stages, are confronted with many challenges, both because of the disease and complications of treatment (Saberzadeh-Ardestani et al ., 2019; Wong et al., 2018). Healthcare providers need to support older cancer patients with a palliative care plan that involves symptom management and psychosocial adaptation (Woodrell et al., 2018; McAteer and Wellbery, 2013) improves patient functioning, (Taylor et al., 2013) and lowers costs (Dalal and Bruera, 2017). While recent randomized clinical trials (RCTs) have shown the benefits of palliative care (Osagiede et al., 2018; Fraser et al., 2019; Collins et al., 2018;Pan et al ., 2019; Abdel-Razeq et al., 2019), little is known about the experiences with palliative care in developing countries such as Iran, where only recently have the growing incidence of cancer and the shortcomings in the quality of care have drawn the attention of relevant authorities (Malayeri, 2017; Rassouli and Sajjadi, 2016). A holistic program for providing palliative care services does not yet exist in Iran, and existing services are inaccessible to many patients (Fallahi et al., 2017). As well as, the lack of an optimal insurance system for end of life care and as well as rate of universal health coverage from the aspect of financial support is troublesome in Iran (Etemadi et al., 2017) meaning that most Iranian patients face challenges and must personally finance their care. A comprehensive understanding of the Iranian palliative care system requires considering the socio-cultural context (Etemadi et al ., 2017). Furthermore,besides the health care team, family support is essential in Iranian society and the familyis expected to be heavily involved in different areas of caring for the patientsuch as; handling the paperwork related to the health care or insurance, providing some grooming and hygiene services etc. Hence, there is a lot of pressure on the families and not addressing the needs of the caregivers could negatively influence the health and care of the patient (Northouse et al ., 2010; Marcotte et al ., 2014). As well as, it is customary for palliative care to be provided by family caregivers, often at home (Mobasher et al., 2013; Hashemian et al., 2017 ). There is little research conducted examining the quality of care and availability of a holistic palliative care approach in Iran. This study aims to describe end-of-life care for older cancer patients in Iran to identify gaps and areas of improvement. 

## Materials and Methods

*Experimentals*

*Setting*

A qualitative descriptive study was performed at three university hospitals in Iran that have palliative care units. 

*Participants*

*Participant Inclusion and Exclusion Criteria*

Participants were eligible providing they were: 1) fluent in Farsi (Persian), 2) willing to participate, 3) diagnosed with late-stage cancer, 4) receiving end-of-life cancer care. Exclusion criteria were unable to communicate clearly (speech or hearing problems), unable to communicate clearly (speech or hearing problems), transferred out of palliative care, diagnosed with cognitive impairments and not feeling well. 

Participants have been selected from diverse cohorts of patients, family member caregivers, nurses, oncologists, social workers, chaplains, and psychologists. Participants were chosen by convenience sampling, they were invited by head-nurses and oncologists of each hospital’s palliative ward and was explained about research (Table 1), and 37 individuals agreed to participate.

*Data collectionprocedures*

Semi-structured interviews using a topic guide were conducted by the first author (ZGh, MSc, PhD student, female) between April 2016 and December 2017. To confirm that the questions addressed the aims of the study, the relationship established prior to study commencement, a pilot interview with a nurse was conducted. After completion, the pilot interview was discussed with an expert in qualitative interview techniques, and due to its valuable content, was included in the study. All interviews were performed in a separate room at the palliative care ward. No other person was present during the interviews. All interviews were face-to-face interviews. Interviews continued until theme was reached saturation and no new theme identified. Each interview lasted for 30-60 minutes, was audio-recorded, transcribed verbatim, and translated into English (Price, 2002). 

*Data analysis*

The data was analyzed using thematic content analysis as described by Braun and Clarke, (2006). The six-phase approach as outlined by Braun and Clarke (2006) was followed: 1) Familiarizing oneself with the data by transcribing (the interview transcriptions were reviewed several times by the members of the research group transcript), reading, and rereading the data, 2) Generating initial codes; 3) Searching for themes 4) Reviewing themes 5) Defining and naming ; and 6) Producing the report. The above-mentioned parameters are the most common measures to achieve rigor in qualitative studies (Guba and Lincoln, 1994). As well as, transcripts were returned to participants for their comments. The consolidated criteria for reporting qualitative research (COREQ) reporting guideline was used to provide a framework for the study (Mollaei et al., 2019). The rigor and validity of the study were used by member check, and also each theme was carried out and agreed by all researchers.

## Results

*Characteristics of Participants*

We showed status number of participants, age range, gender, years of clinical practise range, interview location and marital status in table 3. In total, 37 participants (12 male and 25 female), including 14 nurses, 3 oncologists, 1 social worker, 1 chaplain, 1 psychologist, 11 family members and 6 patients were interviewed. 

*Main Themes*

**Figure 1:**
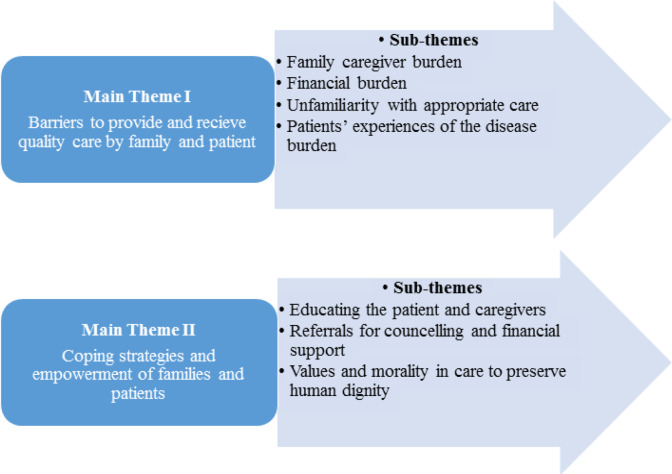
Thematic Map of Study

The qualitative analysis resulted in two main themes of: 1) barriers to providing and receiving quality care for families and patients, and 2) coping strategies and empowerment of families and patients, comprised of seven sub-themes: 1) family caregiver burden 2) financial burden 3) lack of care awareness 4) patients’ experiences of disease burden, and 5) educating the patient and caregivers 6) referrals for counselling and financial support 7) values and morality in care to preserve human dignity (Table 4 and Figure 1).

*Main theme 1: Barriers to providing and receiving quality care by family and patients*

The participants stated that the pressure of caring for patients is too heavy for family caregivers, they experience burnout, takes a lot of time, and were tired. Some of the participants also described physical problems such as physical pain in their shoulders, knees, hands, back, and also headaches they (family caregivers) due to the caregiving process. We extracted the barriers to provide and receive quality care by family and patient and divided them into four sub-themes: 1) Family caregiver burden, 2) Financial burden, 3) Lack of care awareness, 4) Patients’ experiences of disease burden.

*Sub-theme 1.1: Family caregiver burden*

The family is heavily involved in the care and hence their burn out is a barrier to further providing quality care. Multiple participants stated that their family caregivers experience a high level of mental stress that can have a negative impact on their mental health. One participant stated that the family caregivers have to invest a lot of time on providing care, besides their own personal life and jobs, and life is not satisfying for them.

“*Particularly those who care for the patient and are influenced by the patient are in very difficult situation. Have a very difficult life; their lives are severely impacted by them caring for the patient. Everything becomes better, life is not satisfying for them.*” [Female, Social Worker, P36]

“*Different problems arise when a person becomes ill and it affects the whole family of the patient, for example, running around to find medication [since sometimes cancer medications are hard to find in Iran].*” [Daughter, P23 ]

“*There is mental stress involved as well. We [the family members] cannot tell her [the patient] about her illness and we have to be careful because it could have negative impact on her mentality. Because the word ‘cancer’ has a negative connotation. My father was the same, he had cancer but until the last moment of his life he didn’t know he had cancer. We need to consider their morale; and it’s not a good idea for me, to tell him ‘you have cancer’. It’s hard to come into terms with.*” [Son, P20]

*Sub-theme 1.2: Financial burden*

In Iran, not all hospitalization fees are covered by the insurance plan and the costs might be unaffordableor unbearable for the patient that this is direct cost for them.Hospitals try to help by identifying the patients in need and referring them to social workers or charities. The financial burden on patients is much higher for those with end-stage conditions than others (Davari et al., 2013; Rezapour et al., 2018). There are also indirect costs to patients who must travel to receive treatment, such as those living in rural areas. These costs include domestic and international travel fares, hotel accommodations, and food expenses (Mojahedian et al., 2019).The total medical and non-medical fees paid out-of-pocket by patients during treatment was, on average, 25,690,000 Iranian Rials (approximately US $610) per patient during a five-month period. Fifty-three percent of patients took out loans from banks, borrowed money from friends, and appealed to charities to cover their medical costs (Bazyar et al., 2012). 

As expected most of the participants stated that they had financial problems due to their increased burden during the caregiving process. Two of them even stated that they had to sell their belongings or properties to bear the costs. 

“*It has been a financial problemwhile that they have not received insurance payouts. But now they have to pay for themselves. And they’re not doing okay financially.*” [Female, nurse, p10]

*Sub-theme 1.3: Unfamiliarity with appropriate care*

Most of the participants complained about not receiving proper information about the care such as information about the best nutrition for their family member, how to change dressings at home, what to do when a patient has hypotension, even though they had specifically requested it from the healthcare team. This lack of knowledge hinders the family from providing proper care as they do not know what is good for the patient and what is not. 

“*But they don’t give me any information about food. So I don’t know what it is good for him [patient].*” [Daughter, P34]

“*Some of our friends and relatives also want to help, but they don’t have enough information or proper knowledge of the situation. My mother is getting worse. But they tell her, ‘There was this person who had her breast removed and she had chemotherapy and now she’s doing well.You’ll be fine too.’ My mom knows that this is not true for her situation, so she gets upset. She says, ‘why do they talk about something that they don’t know about it…*” [Daughter, P30]

*Sub-theme 1.4: Patients experiences of disease burden*

Most of the respondents emphasized that they (patients) have a lot of concerns and have a wide range of problems. They feel like a burden for their families. They don’t want to be a trouble for others. So sometimes patients refused to eat or drink to avoid having to need using the washroom. So they don’t have to bother their family caregivers as they feel ashamed of need for help. One participant stated that some of the family caregivers didn’t want to accept this burden, this is one of the challenges that patients might face. 

“*When my mom [the patient] is left alone in the home, she feels or expresses distress and annoyance.*” [Daughter, P34]

“*She [the patient] is in limbo. She’s really frustrated. Because her body also hurts.*” [Daughter, p30] 

The patient told me” *I’m just tired. Tired of extreme pity*” ... caregivers should avoid extreme pity. [Female, psychologist, P35].

*Main theme 2: Coping strategies and empowerment of families and patients*

Coping strategies are the ability to manage threatening and challenging situations for supporting families and patients. The theme coping strategies and empowerment of families and patients is grouped in three sub-themes: 1) educating the patient and caregivers, 2) referrals for counselling and financial support, and 3) values and morality in care to preserve human dignity.

*Sub-theme 1.1 Educating patients and caregivers*

Some of the participants stated that health care providers should provide training and educate for patients and their family caregivers about the situation of the patients and their care. Such as, they should know about the side effects of their medication. It’s important that proper education and information is provided for patients and family caregivers. The healthcare team encourage families to take turns so the patient is not taken care of by only one person, but everyone shares the responsibility. In Iran, patients’ families are expected to be active in the patient’s care. Although this is not an official rule, it is an accepted convention that families must be availableto provide water for patients, fill prescriptions, collect test results, etc. They are also needed as next-of-kin to provide consent where necessary.

“*It is important to teach the family members to share responsibilities such as encourage families to take turns so the patient is not taken care by only one person…. We have to conduct meetings and conferences for families to update them on their [the patient’s] condition.” “To teach the family how to care for the patient…To educate the family to avoid pitying the patient...* [Female, Psychologist, P35] 

*Sub-theme 1.2: Referrals for counseling and financial support*

Some of the participants stated that they were referred to social workers to receive financial and counselling support to psychology and also refer to social worker and charitable organizations that even provided a place to stay for patients and their family members. They (health care team) spoke to patients and their family caregivers and provided some supports for them.

“*They [patients or their family members] talk about their troubles and pain. … And if their mental health becomes worse, we will send them to talk to a psychologist about their problems.*” [Female, nurse, p1]

“*There are times when both patients and their family members have greater need for mental support, because the process prolongs further than expected. They can be referred for counselling*” [Female, head nurse, p12]

*Sub-theme 1.3: Values and morality in care to preserve human dignity*

Nurses indicated that it is important to listen to patient preferences and maintain patient’s dignity. Because if a patient realizes that she/he does not have time, can do some of the things that she/he has always wished to do such as traveling to a certain place, that thus they should be provided more information about their condition and make them feel comfortable.

“*The best care for the patient is being honest with them. In my opinion, it should be embedded in the framework of patient care as the patient needs it. If a patient suspects dishonesty from the healthcare team, she/he cannot trust them to receive care.*” [Male, nurse, p11]

“*They [the family members] were tired of being caregivers. They came here and said, ‘Now, while she [the patient] is admitted here, we can rest a bit, and rebuild our strength, and we can take care of her when she returns home…. It is important to pay attention to the caregiver’s demands.*’” [Female, nurse, p1]

## Discussion

Two main themes emerged from our content analysis identifying end of life care for cancer patients and accomplishing the best care for them from their care providers. One such theme identifies barriers to provide and receive quality care by family and patient. Due to the long durationand due to their involvement, also they reported that the patients and their family caregivers experience health problems and burden.Several studies have reported in Iran high burden, family distress, anxiety and death anxiety, lifestyle disruption, unmet needs of the family caregivers, lack of special education, confusion, uncertainty, financial problems, lack of providing information, which is in line with our findings (Naghavi et al., 2009; TorabiParizi et al., 2018). Furthermore, similar to our study, several studies outside of Iran have reported fatigue, sleep disturbance, anxiety, depression, loss of control, decreased satisfaction, functional impairment, physical pain, financial burden, lack of knowledge and fatigue (TorabiParizi et al., 2018; Ziemba , 2016 ; Johnson et al., 2007; Parmar et al., 2014; Rose et al., 2004). In addition to, the disclosure of the disease in Iran is not culturally possibleand due to the lack of disclosure to patients and their families mental stress of caregiving is higher (TorabiParizi et al., 2018). Furthermore, patients and their families must finance their care through insurance and out-of-pocket payments (Mollaei et al., 2019; Davari et al., 2013 ) which compared to research outside of Iran shows that in Iran we need to more attention for the financial part at the end of life for cancer patients particular in older adults (Bélanger et al., 2018; Bansal , 2018). In addition to, there are still, very limited financial resources (Kennedy, 2016), inadequate resources (Walshe et al., 2017) and lack palliative-care services in developing countries (Terzioglu and Hammoudeh, 2017) and need to more suitable health financing system (Messinger et al., 2017).

The other main theme focused on coping approaches for empowerment of families and patients. Similar to our study, several studies have reported coping with stress, education, referrals them to counselling and motivate them and financial support and respect to their values and dignity in them by attention to their needs and led to reducing stress and worrying (Ashrafian et al., 2018; Cruz-Oliver et al., 2017; Yousefinezhadi et al., 2017; Johansen et al., 2018 ). Also, several studies have reported that families play an important role in the daily care for patients admitted on the palliative care unit (Naghavi et al., 2009; Cruz-Oliver et al., 2017). 

The current study is included 37 participants, which represents a small sample size, and only comprised three teaching hospitals in Iran; thus, the findings from this study cannot be generalised, and they only reflect the experiences of this group of participants. And for future research, both burdens and copings topic especially study of financial burden are high priorities for future researches with large sample size. 

The current study is included 37 participants, which represents a small sample size, and only comprised three teaching hospitals in Iran; thus, the findings from this study cannot be generalised, and they only reflect the experiences of this group of participants. And for future research, both burdens and copings topic especially study of financial burden are high priorities for future researches with large sample size.

In conclusion, healthcare providers are recommended to familiarize themselves with the burden faced by patients and family caregivers who take care of older adults with chronic diseases at home, and they should organize their supportive and consulting actions according to family situations in order to improve the quality of life of older patients and their family caregivers.In this study, families indicated a need to pay more attention to their needs. There is therefore a need for greater support services for patients receiving home care especially for patients with end-stage diseases. 

**Table 1:** Participant Characteristics

Participants	Number of participants	Age range	Gender	Years of clinical practise range	Marital status
Nurses	14	34-58	80% Female	10-35	85% Married
			20% Male	-	15% Single
Patients (cancer: colorectal, stomach, abdominal)	6	50-83	50% Female	3-10	100% Married
			50% Male	-	
Medical specialists/ oncologists	3	34-45	100% Male	6	100% Married
Family members (sons, daughters, spouses)	11	32-67	80% Female	3	90% Married
			20% Male	8	10% Single
Psychologists	1	30	100% Female	6	100% Single
Chaplains	1	33	100% Male	3	100% Married
Social workers	1	40	100% Female	8	100% Single
